# Novel *Ehrlichia* Strain Infecting Cattle Tick *Amblyomma neumanni*, Argentina, 2018

**DOI:** 10.3201/eid2605.190940

**Published:** 2020-05

**Authors:** Lucía Fargnoli, Camilo Fernandez, Lucas D. Monje

**Affiliations:** Instituto de Ciencias Veterinarias del Litoral, UNL-CONICET, Esperanza, Argentina

**Keywords:** *Ehrlichia*, *Amblyomma neumanni*, cattle, Chaco Seco, Argentina, rickettsia, zoonoses, vector-borne infections, ticks

## Abstract

In 2018, we detected a novel *Ehrlichia* strain infecting *Amblyomma neumanni* ticks in Argentina. The novel strain is phylogenetically related to the ruminant pathogen *E. ruminantium* and represents a potential risk for veterinary and public health because *A. neumanni* ticks parasitize domestic and wild ruminants and bite humans.

*Ehrlichia* spp. are intracellular gram-negative bacteria relevant to human and animal health; they infect monocytes, neutrophils, or endothelial cells, depending on the species involved (*1*). The genus *Ehrlichia* (Rickettsiales: Anaplasmataceae) comprises 6 formally recognized tick-transmitted species: *E. canis*, *E. muris, E. chaffeensis*, *E. ewingii*, *E. minasensis*, and *E. ruminantium* (*2*,*3*). Recently, other *Ehrlichia* species have been reported and diﬀerent strains of putative novel *Ehrlichia* species have been molecularly detected, but their taxonomic positions are still not clearly defined (*4*–*6*). Current knowledge about this group of pathogens suggests that a large number of *Ehrlichia* species might be not yet described.

*Amblyomma neumanni* ticks are relevant to human and veterinary medicine because in all stages they commonly parasitize wild and domestic ruminants and other large mammals, including humans (*7*). Moreover, *A. neumanni* ticks can reportedly be infected with *Rickettsia bellii* and *Rickettsia amblyommatis* and are potential vectors of the cattle pathogen *Anaplasma marginale* (*7*). To determine the presence of tickborne bacteria of the genus *Ehrlichia* in questing *A. neumanni* ticks in northwestern Argentina, we performed phylogenetic analyses on Anaplasmataceae-positive tick DNA samples. All procedures were approved by the Ethics and Biosafety Committee of the Facultad de Ciencias Veterinarias, Universidad Nacional del Litoral, Esperanza, Argentina.

During May 2018 (late autumn), we collected free-living ticks by dragging and by using dry ice-baited traps in Dean Funes (30°22′S, 64°21′W) and San José de la Dormida (30°21′S, 63°58′W), Córdoba Province, Argentina. Both sites are located in the Chaco Seco ecoregion. We identified all ticks by using standard taxonomic keys (*7*) and individually processed them for DNA extraction by using a boiling method (*8*). We screened DNA extracts for Anaplasmataceae by real-time PCR targeting the 16S rRNA gene, as previously described (*9*). We further tested samples positive for Anaplasmataceae by amplification of *Ehrlichia* genes *dsb* and *groESL*, as described elsewhere (*5*). We sequenced all amplicons and performed phylogenetic analyses with the maximum-likelihood method.

We collected 229 ticks from Dean Funes (70 adults, 159 nymphs) and 62 from San José de la Dormida (24 adults, 38 nymphs) and identified all ticks as *A. neumanni*. Only 1 adult tick from San José de la Dormida was positive for Anaplasmataceae by PCR. Further analysis of that sample resulted in 2 sequences of 355 bp (*dsb*) and 784 bp (*groESL*). Phylogenetic analysis of the *dsb* sequence (GenBank accession no. MN176580) showed that the *A. neumanni* tick was infected with a species of *Ehrlichia*, which we named *Ehrlichia* sp. strain La Dormida, closely related to *E. ruminantium* (82.0%, GenBank accession no. CR925677) (Figure, panel A). Other ehrlichiae from South America included in the analysis, such as *Ehrlichia* sp. strain Córdoba from Argentina (78.0%, GenBank accession no. KY413807) and *Ehrlichia* sp. strain Natal from Brazil (79.3%, GenBank accession no. KY207546), were placed in a clade sister to the group formed by *Ehrlichia* sp. strain La Dormida and *E. ruminantium*. Furthermore, the phylogenetic analysis performed by using the *groESL* sequence (GenBank accession no. MN176581) confirmed these results (Figure, panel B). 

**Figure:**
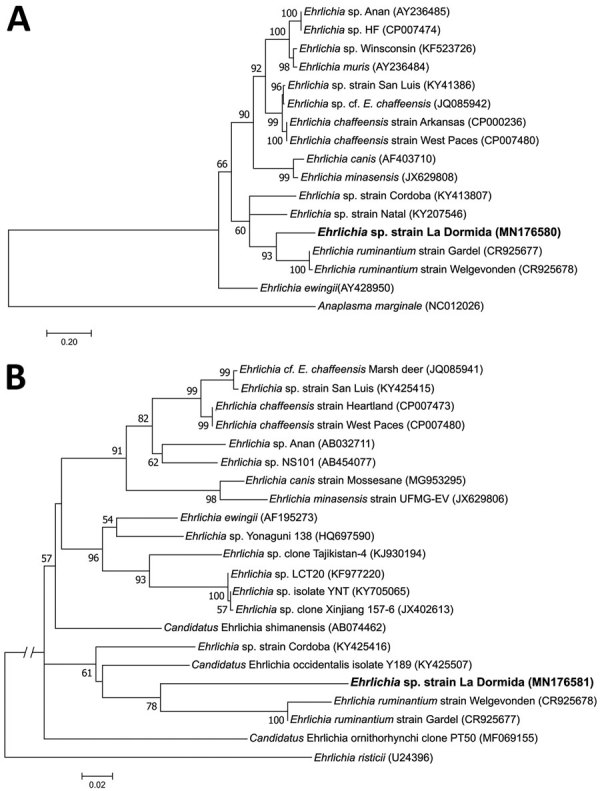
Maximum-likelihood trees constructed from *dsb* and *groESL* sequences of *Ehrlichia* sp. infecting *Amblyomma neumanni* ticks in Argentina compared with reference strains. A) Tree constructed by using *dsb Ehrlichia* sequences of approximately the same length as the sequence identified in this study (341 positions included in the final dataset). B) Tree constructed by using *groESL Ehrlichia* sequences of approximately the same length as the sequence identified in this study (767 positions included in the final dataset). Phylogenetic trees were constructed by using MEGA 7.0 (https://www.megasoftware.net), and best-fitting substitution models were determined with the Akaike Information Criterion, using the maximum-likelihood model test. Numbers represent bootstrap support generated from 1,000 replications. GenBank accession numbers are shown in parentheses. Boldface indicates the strain identified in this study. Scale bars indicate nucleotide substitutions/site.

Several recent studies conducted in South America reported finding novel ehrlichial agents infecting jaguars, horses, crab-eating foxes, opossums, sloths, and peccaries (*6*). Unfortunately, only short *dsb* sequences are available for those ehrlichiae from South America. Phylogenetic analysis including these sequences (210 positions included in the final dataset) placed them all together in a clade sister to the group formed by *Ehrlichia* sp. strain La Dormida and *E. ruminantium* (Appendix Figure).

*Ehrlichia* sp. strain La Dormida, associated with *A. neumanni* ticks, circulates in rural areas of northwestern Argentina. In our phylogenetic analyses, *Ehrlichia* sp. strain La Dormida genotype was unique and well separated from all other available ehrlichial sequences, suggesting that it could represent a distinct species yet to be properly characterized. In addition, these analyses positioned *Ehrlichia* sp. strain La Dormida in a separate group together with *E. ruminantium*. The species *E. ruminantium* is native to the Africa continent, where it is the etiologic agent of heartwater, a tickborne disease of major economic relevance with regard to domestic ruminants throughout sub-Saharan Africa (*10*). Besides *E. ruminantium*, the only other species of *Ehrlichia* known to naturally infect and cause clinical manifestations of ehrlichiosis in cattle is *E. minasensis* (*3*). However, *E. minasensis* is phylogenetically distant from *E. ruminantium* and closely related to *E. canis* genotypes (*3*). 

We report *A. neumanni* ticks as a potential vector of *Ehrlichia* sp. strain La Dormida. Because it is assumed that bacteria of the genus *Ehrlichia* are not transmitted transovarially in ticks (*1*), infection with *Ehrlichia* must be acquired during feeding of immature ticks, which then pass the infection to adults by transstadial transmission. Regarding *E. ruminantium*, wild African ruminants are reservoirs of the bacteria (*10*). The novel *Ehrlichia* sp. strain La Dormida is phylogenetically related to the ruminant pathogen *E. ruminantium* and represents a potential risk for veterinary and public health because *A. neumanni* ticks parasitize domestic and wild ruminants and bite humans.

Appendix Phylogeny of *Ehrlichia* spp. from South America.
