# Supplementary material for: Novel Ehrlichia Strain Infecting Cattle Tick Amblyomma neumanni, Argentina, 2018
Source: Emerg Infect Dis. 2020 May;26(5):1027–30. doi: 10.3201/eid2605.190940 (PMC7181906; doi:10.3201/eid2605.190940)
Supplement: Appendix — Phylogeny of Ehrlichia spp. from South America. [file 19-0940-Techapp-s1.pdf]

Article DOI: https://doi.org/10.3201/eid2605.190940

Novel *Ehrlichia* Strain Infecting Cattle Tick *Amblyomma neumanni*, Argentina, 2018

Appendix

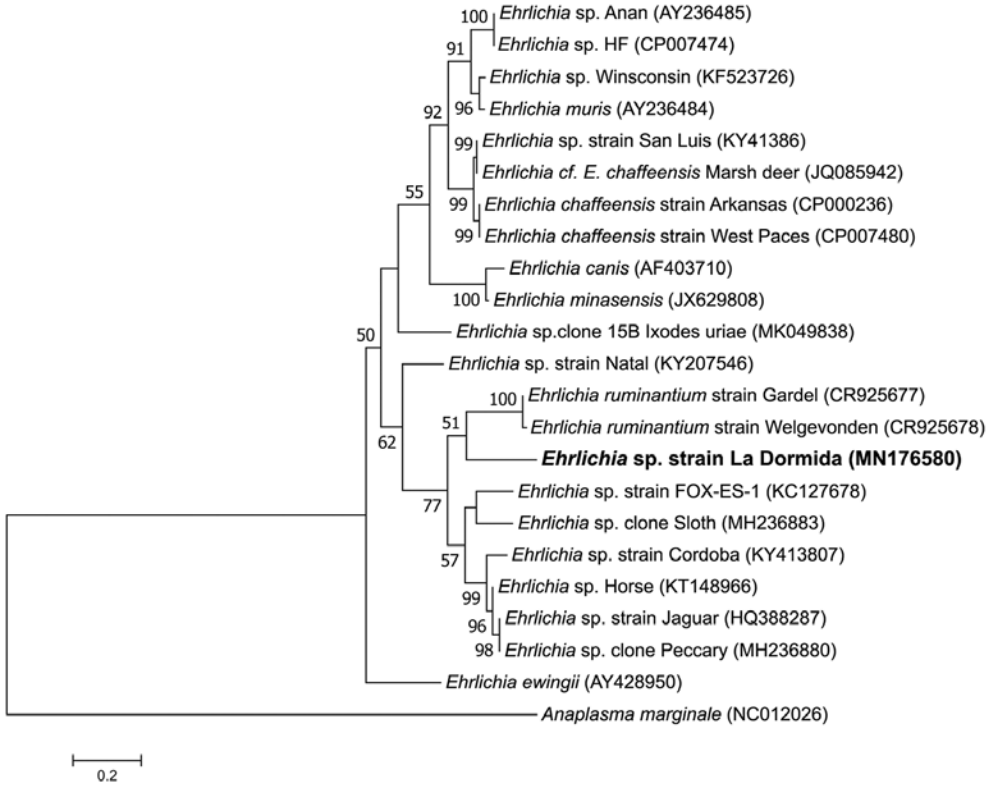

Appendix Figure. Maximum Likelihood tree constructed by using shorter *dsb* sequences reported from other South American *Ehrlichia* sp. (210 positions included in the final dataset). Phylogenetic tree was constructed by using the program Mega 7.0 (https://www.megasoftware.net/) and best fitting substitution models were determined with the Akaike Information Criterion using the ML model test. Numbers represent bootstrap support generated from 1000 replications. GenBank accession numbers are in brackets.
